# Low-Dose Stevia (Rebaudioside A) Consumption Perturbs Gut Microbiota and the Mesolimbic Dopamine Reward System

**DOI:** 10.3390/nu11061248

**Published:** 2019-05-31

**Authors:** Jodi E. Nettleton, Teja Klancic, Alana Schick, Ashley C. Choo, Jane Shearer, Stephanie L. Borgland, Faye Chleilat, Shyamchand Mayengbam, Raylene A. Reimer

**Affiliations:** 1 Faculty of Kinesiology, University of Calgary, 2500 University Drive NW, Calgary, AB T2N 1N4, Canada; jenettle@ucalgary.ca (J.E.N.); teja.klancic@ucalgary.ca (T.K.); acchoo@ucalgary.ca (A.C.C.); jshearer@ucalgary.ca (J.S.); fatima.chleilat1@ucalgary.ca (F.C.); shyamchandsingh.maye@ucalgary.ca (S.M.); 2 International Microbiome Centre, Cumming School of Medicine, University of Calgary, 3300 Hospital Drive NW, Calgary, AB T2N 4N1, Canada; a.schick@ucalgary.ca; 3 Department of Biochemistry and Molecular Biology, Cumming School of Medicine, University of Calgary, 3300 Hospital Drive NW, Calgary, AB T2N 4N1, Canada; 4 Hotchkiss Brain Institute, University of Calgary, 3300 Hospital Drive NW, Calgary, AB T2N 4N1, Canada; slborgla@ucalgary.ca

**Keywords:** low-calorie sweeteners, stevia, Rebaudioside A, gut microbiota, glucose tolerance, mesolimbic reward system, oligofructose-enriched inulin

## Abstract

Stevia is a natural low-calorie sweetener that is growing in popularity in food and beverage products. Despite its widespread use, little is understood of its impact on the gut microbiota, an important environmental factor that can mediate metabolism and subsequent obesity and disease risk. Furthermore, given previous reports of dysbiosis with some artificial low-calorie sweeteners, we wanted to understand whether prebiotic consumption could rescue potential stevia-mediated changes in gut microbiota. Three-week old male Sprague–Dawley rats were randomized to consume: (1) Water (CTR); (2) Rebaudioside A (STV); (3) prebiotic (PRE); (4) Rebaudioside A + prebiotic (SP) (*n* = 8/group) for 9 weeks. Rebaudioside was added to drinking water and prebiotic oligofructose-enriched inulin added to control diet (10%). Body weight and feces were collected weekly and food and fluid intake biweekly. Oral glucose and insulin tolerance tests, gut permeability tests, dual X-ray absorptiometry, and tissue harvest were performed at age 12 weeks. Rebaudioside A consumption alone did not alter weight gain or glucose tolerance compared to CTR. Rebaudioside A did, however, alter gut microbiota composition and reduce nucleus accumbens tyrosine hydroxylase and dopamine transporter mRNA levels compared to CTR. Prebiotic animals, alone or with Rebaudioside A, had reduced fat mass, food intake, and gut permeability and cecal SCFA concentration. Adding Rebaudioside A did not interfere with the benefits of the prebiotic except for a significant reduction in cecal weight. Long-term low-dose Rebaudioside A consumption had little effect on glucose metabolism and weight gain; however, its impact on gut microbial taxa should be further examined in populations exhibiting dysbiosis such as obesity.

## 1. Introduction

The global rate of obesity has increased dramatically in the past 30 years, and it is now estimated that nearly 650 million adults and 124 million children and adolescents worldwide are living with this chronic disease [1]. Obesity, although complex in its etiology, results from a positive energy balance, where more energy is consumed than is expended in physical activity, resting metabolic rate, and the thermic effect of food [2]. Additionally, there has been steady and rapid growth of the availability and consumption of a “Western” diet with calorically dense food and beverage products high in fat and sugar [3]. Foods that characterize the Western diet are becoming increasingly available in developing countries alongside growing overweight and obesity rates [4].

To reduce the caloric content of certain food and beverage products, low-calorie sweeteners (LCS) are becoming increasingly popular and can be found in food products typically labelled “lite”, “diet”, and “sugar-free”. There has been growing interest in and consumption of naturally occurring sweeteners, like stevia, perhaps in part due to consumer perception of the benefits and lack of risk around natural foods [5,6]. The sweetener stevia is found in perennial shrub species *Stevia rebaudiana* Bertoni, which is indigenous to Central and South America [7], and is approximately 200–400 times sweeter than sucrose on a weight basis [8].

Historically, stevia has been therapeutically used in Paraguay and Brazil to treat diabetes [9], and studies have highlighted stevioside’s ability to normalize blood glucose levels in humans with diabetes and in diabetic rodent models [10,11,12]. Ahmad et al. (2018) observed that diabetic rats (streptozotocin-induced) consuming stevia for 8 weeks had reduced calorie and fluid intake, lower blood glucose levels and body weight, and increased insulin and liver glycogen in a dose-dependent manner [13]. Although most studies have found a positive impact of stevia glycosides on metabolic parameters, doses have greatly exceeded the adequate daily intake [12] recommended by governing health agencies like Health Canada and the US Food and Drug Administration from ten- to one-hundred-fold [8,14].

To date, the majority of research examining the impact of stevia on metabolic health has examined a single bolus or short-term delivery of the additive. There is a growing field of literature showing that chronically consuming low-dose LCS may promote weight gain [15], reduce glucose tolerance and insulin sensitivity [16], and interfere with brain regions that play a critical role in appetitive behaviour (Nettleton et al., personal communication) and gut microbiota composition [17], suggesting LCS have a greater impact on metabolic health than previously believed. To date, the majority of the evidence for metabolic disruptions has been demonstrated with artificial low-calorie sweeteners, and more work is needed with naturally-derived, plant-based sweeteners such as stevia.

Gut microbiota is a well-established and vital contributor to health, and disruptions in gut microbial communities, or dysbiosis, play a role in the development of chronic diseases, including obesity [18]. LCS consumption changes gut microbiota composition [16,17] and this microbial alteration has been found to play a causal role in the adverse health outcomes observed [17]. For example, Suez and colleagues observed glucose intolerance in lean and obese mice and human participants fed saccharin, and fecal microbiota transplant transferred this adverse phenotype to germ-free mice [17]. Palmnäs et al. found that mice consuming aspartame had increased abundance of *Enterobacteriaceae* and *Clostridium leptum* alongside altered insulin-mediated glucose clearance [16]. More recently, mice consuming sucralose and chow displayed a significant increase in Firmicutes and reduction in Bacteroidetes abundance [19], highlighting the capacity of different LCS to alter gut microbiota composition and mediate aberrant metabolic outcomes. Stevia compounds are not digested by the host but are metabolized by microbiota and absorbed into circulation, where it is ultimately excreted in urine [20]. Stevia compounds therefore have the potential to interact with and alter microbial communities in the colon. In fact, Rebaudioside A, a major stevia glycoside, significantly reduced the growth of *Escherichia coli* strain HB101 on agar plates [19]. Thus, our objective was to examine the impact of chronic, low-dose consumption of stevia on body composition, glucose tolerance, and insulin sensitivity and the potential role of gut microbiota in mediating these changes. We hypothesized that potential adverse effects stemming from stevia consumption may be rescued by consuming a prebiotic, a substrate that is selectively utilized by host microorganisms conferring a health benefit [21]. Prebiotics, particularly chicory root-derived oligofructose and inulin, have been shown to rescue phenotypes induced by poor diet, such as obesity and gut microbiota dysbiosis [22,23]. In addition, there is a growing demand for functional foods that target weight control and reduce symptoms of the metabolic syndrome; therefore, examining the combination of two ingredients is important in understanding their impact on metabolic health when consumed alone or in combination.

## 2. Materials and Methods

At age 3 weeks, male Sprague–Dawley rats consuming an AIN-93G (Dyets^®^, Bethlehem, PA) control diet were randomized to one of four groups for 9 weeks: (1) Water (CTR; control group); (2) Rebaudioside A (STV; 2–3 mg/kg; Rebaudioside A, Sigma, Oakville, ON, Canada); (3) prebiotic (PRE; 10% wt/wt oligofructose-enriched inulin, Synergy1, Beneo, Mannheim, Germany); or (4) prebiotic + Rebaudioside A (SP; 2–3 mg/kg Rebaudioside A + 10% oligofructose-enriched inulin). Rebaudioside A (RebA) was administered through the drinking water and prebiotic was administered in the food. The glycoside Rebaudioside A was chosen as it is one of the most abundant glycosides contributing to the sweet taste of stevia and has a significantly reduced bitter aftertaste. The composition of the experimental diets is found in Supplementary Table S1 and S2. Food and fluid intake were recorded every second week for five consecutive days. Insulin tolerance tests (ITT), oral glucose tolerance tests (OGTT), and gut permeability tests were performed at age 11 weeks, with 48 h separation between tests. Ethical approval was granted by the University of Calgary Animal Care Committee (Protocol #AC15-0079) and conformed to the Guide to the Care and Use of Laboratory Animals.

### 2.1. Insulin Tolerance Test

Following a 6-hour fast, rats received an insulin load (0.75 U/kg) by intraperitoneal injection. Glucose concentrations were measured immediately in blood from the tail vein with a One Touch^®^ Ultra^®^ 2 glucose meter (LifeScan, Burnaby, Canada) at baseline (fasting), and 15, 30, 60, 90, and 120 min post-injection.

### 2.2. Oral Glucose Tolerance Test

Following an overnight fast, rats received an oral glucose load (2 g/kg) via oral gavage. Blood glucose concentrations were measured at baseline (fasting), and 15, 30, 60, 90, and 120 min post-gavage via tail vein using a One Touch^®^ Ultra^®^ 2 glucose meter.

### 2.3. Gut Permeability Test

Following a 6-hour fast, rats were gavaged with fluorescein isothiocyanate–dextran-4000 (DX-4000-FITC; 250 mg/kg, 125 mg/mL). Blood was collected via tail vein 1-hour post-gavage in a tube with ethylenediaminetetraacetic acid (EDTA) and stored on ice and kept in the dark. Samples were centrifuged at 4 °C for 3 min at 12,000 g and stored at −80 °C until analysis. Plasma samples were diluted in equal volumes of PBS, and 50 µL were loaded in duplicate onto a 96-well plate alongside serially diluted standards. The plate was read on a microplate fluorescence reader (FLX 800) at an excitation wavelength of 485 nm and emission wavelength of 535 nm.

### 2.4. Body Composition and Tissue Collection

At age 12 weeks, rats underwent a dual X-ray absorptiometry (DXA) scan under light anesthesia (isoflurane) using Hologic QDR software for small animals (Hologic ODR 4500; Hologic Inc., Bedford, MA, USA). Lean and fat mass (g), body fat (%), and bone mineral density (g/cm^2^) were recorded. Following overnight feed deprivation, rats were overanesthetized with isoflurane and killed by decapitation. Liver and cecum were excised and weighed and cecal matter and colon were collected, snap frozen, and stored at −80 °C until analysis. Shortly after decapitation, the brain was excised, and the ventral tegmental area and nucleus accumbens were isolated, snap frozen, and stored at −80 °C for subsequent analysis.

### 2.5. Microbiota Sequencing

Cecal matter was collected posthumously, snap frozen, and stored at −80 °C. Bacterial genomic DNA was extracted using FastDNA spin kit for feces (MP Biomedicals, Lachine, QC, Canada) and sequenced by the Centre for Health Genomics and Informatics at the University of Calgary on the Illumina MiSeq platform, sequencing the 16S hypervariable V3–V4 regions as previously described [24]. All sequence analysis was performed in R, version 3.5.2. Raw sequence reads were filtered for quality using the R package dada2, version 1.10.1 [25]. A table of amplicon sequence variants (ASVs) was generated using dada2 and taxonomic classifications assigned using the Silva 132 database as a reference. Using the R package phyloseq, version 1.24.2 [26], alpha-diversity was estimated by calculating the Chao1, Shannon, and Simpson indices. Beta-diversity was evaluated using nonmetric multidimensional scaling (NMDS) on a Bray–Curtis dissimilarity matrix. An analysis of differentially abundant features between groups was carried out using LEfSe [27], with a significance of alpha = 0.05.

### 2.6. Short Chain Fatty Acid Analysis

The concentration of short chain fatty acids (SCFAs) in cecal matter was measured using HPLC and an internal standard of 2-ethyl butyric acid. Fatty acid derivatization was performed as previously described [28], with modifications. SCFAs contained in cecal matter (250 mg) were extracted in 500 uL of 0.15 mmol/L H_2_SO_4_ containing internal standard (2-ethyl butyric acid) by homogenizing for two 40 s cycles using FastPrep-24^TM^ homogenizer (MP Biomedicals, Santa Anna, CA, USA). Samples were then centrifuged at 14,000 g for 15 min at 4 °C and supernatant collected. Fatty acid derivatization was performed as previously described, with modifications [28]. Briefly, 100 µL of aqueous extract was transferred to a reaction tube, and 200 µL of each of 3-nitrophenylhydrazine (20 mmol/L), 3% Pyridine, and 1-ethyl-3-(3-dimethylamineopropyl) carbodiimide (250 mmol/L) were added. The mixture was incubated at 60 °C for 20 min. After the addition of 100 uL of KOH solution, the mixture was further heated at 60 °C for another 15 min. The sample was cooled at room temperature and then mixed with 4 mL phosphate buffer mixture (0.03 mol/L phosphate buffer, pH 6.4 and 0.5 mol/L HCl at 3.8:0.4 v/v). A total of 3 mL of hexane was added, tubes were vortexed, and the supernatant was discarded; 3 mL of diethylether was added to the mixture and SCFA derivatives isolated on a shaker for 10 min. The supernatant was dried in a speedvac concentrator (Savant™ SPD111 SpeedVac™ Kits, Thermo Scientific™, Waltham, MA, USA). The residue was dissolved in 1 mL of 50% methanol and centrifuged at 14,000 g for 15 min at 4 °C. The clear supernatant was collected and stored in −20°C until assayed. A total of 20 µL of the sample was injected in reverse-phase HPLC on a C18 column containing a column guard. The sample was eluted in a gradient of acetonitrile containing 0.05% trifluoroacetic acid (8%–100%), with a flow rate of 0.8 mL/min for 30 min. The absorbance of the eluate was analyzed at 230 nm.

### 2.7. Statistics

All data are mean ± SEM unless otherwise stated. A 2-way ANOVA was used to determine the main effects of RebA and prebiotic, as well as whether an interaction between RebA and prebiotic existed. If a significant interaction between RebA and prebiotic was identified, a one-way ANOVA with all four treatments was performed with Tukey’s post-hoc test to determine differences across all experimental groups. For measurements with repeated measures, a 2-way repeated measures ANOVA was used with time as the within-subject factor and RebA and prebiotic as between-subject factors. Results were considered significant at *p* ≤ 0.05. Statistics were performed with IBM^®^ SPSS Statistics version 24.0.

## 3. Results

### 3.1. RebA Does Not Affect Adiposity but Blunts Prebiotics Ability to Increase Cecal Weight

**Figure 1:**
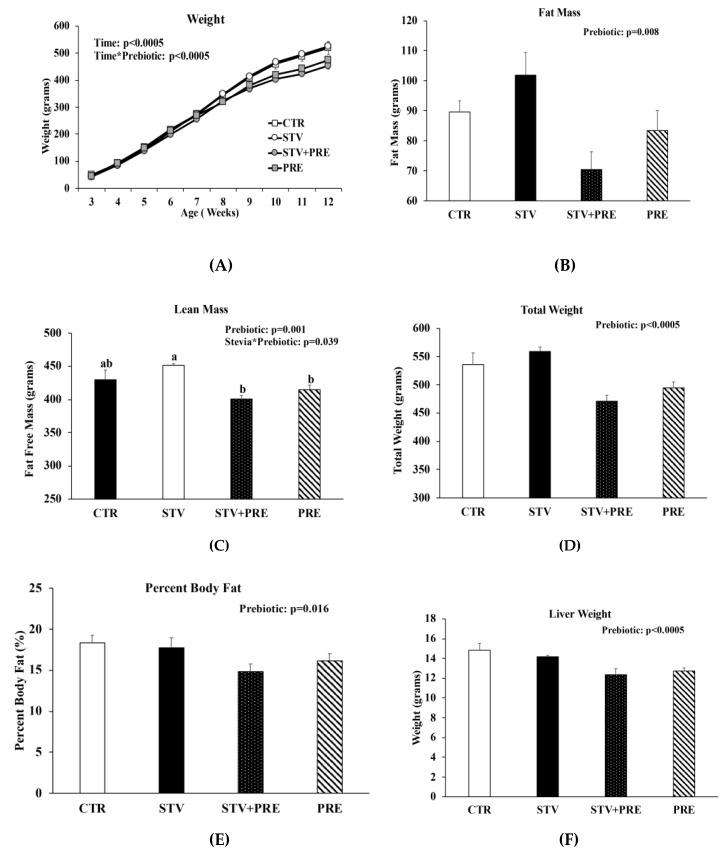

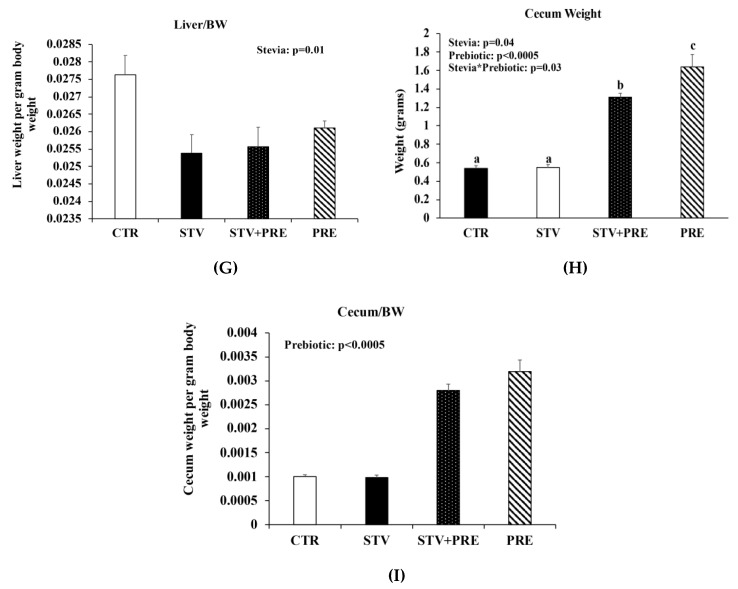
Body weight, body composition, and cecum weight in male rats consuming RebA, prebiotics, both or neither for 9 weeks. (**A**) Body weight; (**B**) fat mass; (**C**) lean mass; (**D**) final total body weight; (**E**) percent body fat; (**F**) liver weight; (**G**) liver weight per gram of body weight; (**H**) cecum weight; (**I**) cecum weight per gram of body weight. Panels B-I were measured at 12 weeks of age. Values are mean ± SEM, *n* = 8/group. Labelled means without a common superscript letter (a, b, c) differ, *p* < 0.05. CTR, control; STV, RebA; STV + PRE, RebA and prebiotic; PRE, prebiotic; BW, body weight.

Body weight increased in all rats over the 9 weeks as they matured, but only prebiotic interacted significantly with time to reduce body weight over the 9 weeks (*p* < 0.0005) (Figure 1A). Fat mass was significantly influenced by prebiotic alone (*p* = 0.008) (Figure 1B), whereas lean mass was influenced by prebiotic (*p* = 0.001) and the interaction between prebiotic and RebA (*p* = 0.039) (Figure 1C). There was a main effect of prebiotic in reducing final total body weight (*p* < 0.0005; Figure 1D), percent body fat (*p* = 0.016; Figure 1E), and liver weight (*p* < 0.0005; Figure 1F). However, RebA consumption significantly reduced liver weight when calculated as liver weight per gram of body weight (Figure 1G). Cecum weight was affected by RebA (*p* = 0.04), prebiotic (*p* < 0.0005), and their interaction (*p* = 0.03) (Figure 1H). Consuming prebiotics markedly increased cecum weight by a mean of 1.1 grams (*p* < 0.0005) (Figure 1H) compared to rats not consuming prebiotics; however, consuming RebA with the prebiotic reduced cecum weight by a mean 0.33 grams (*p* = 0.004) compared to rats consuming only prebiotic. When cecum weight was calculated per gram of body weight, prebiotic significantly increased cecum weight (Figure 1I). There was no significant difference in cecum weight between CTR and STV rats.

### 3.2. Food and Fluid Intake Not Impacted by RebA Consumption

**Figure 2:**
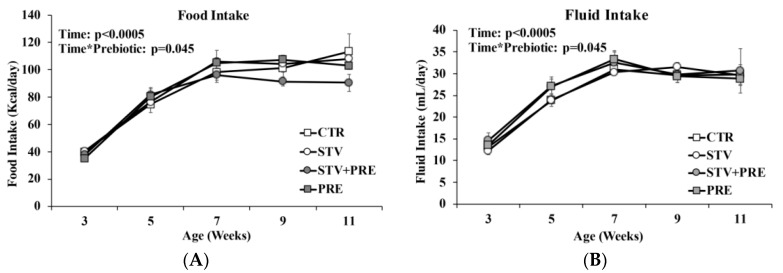
Food and fluid intake in male rats consuming RebA, prebiotics, both or neither for 9 weeks. (**A**) Food intake; (**B**) fluid intake. Values are mean ± SEM, *n* = 8/group. CTR, control; STV, RebA; STV + PRE, RebA and prebiotic; PRE, prebiotic.

Food and fluid intake increased in all rats over time (*p* < 0.0005) (Figure 2A,B). Prebiotic but not RebA reduced food intake as rats grew from 3 to 11 weeks of age (*p* = 0.045).

### 3.3. Prebiotic but Not RebA Improves Insulin Sensitivity

**Figure 3:**
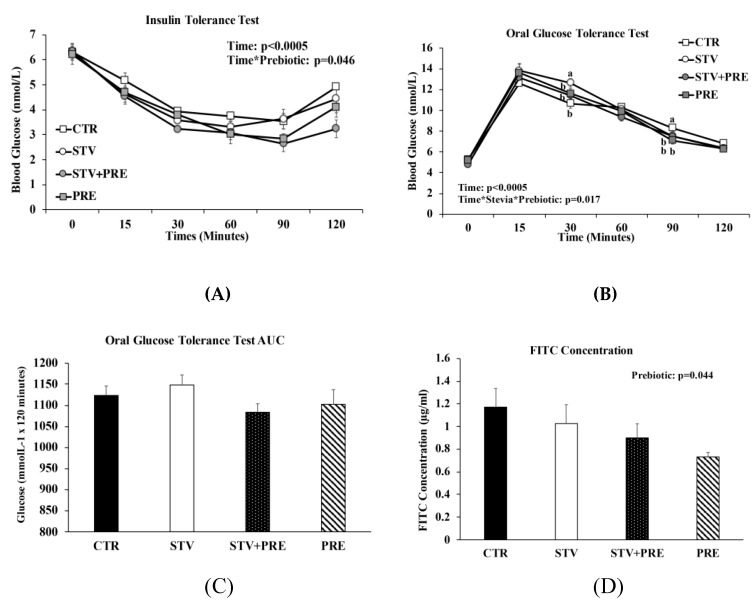

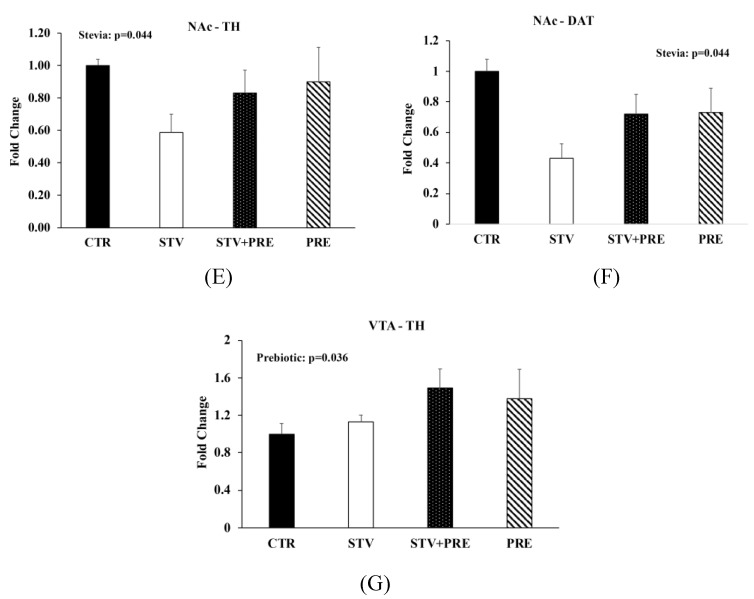
Insulin and glucose tolerance tests, intestinal permeability, and gene expression in the brain of male rats consuming RebA, prebiotics, both or neither for 9 weeks. (**A**) Insulin tolerance test; (**B**) oral glucose tolerance test; (**C**) oral glucose tolerance test area under the curve (AUC); (**D**) gut permeability (FITC) test; (**E**) nucleus accumbens tyrosine hydroxylase mRNA levels; (**F**) nucleus accumbens dopamine transporter mRNA levels; (**G**) ventral tegmental area tyrosine hydroxylase mRNA levels. Values are mean ± SEM, *n* = 8/group. Labelled means without a common superscript letter (a, b, c) differ, *p* < 0.05. CTR, control; STV, RebA; STV + PRE, RebA and prebiotic; PRE, prebiotic; AUC, area under the curve; FITC, fluorescein isothiocyanate–dextran-4000; NAc-TH, nucleus accumbens tyrosine hydroxylase; NAc-DAT, nucleus accumbens dopamine transporter; VTA-TH, ventral tegmental area tyrosine hydroxylase.

As expected, time significantly influenced blood glucose concentration in all rats during the ITT and OGTT (*p* < 0.0005) (Figure 3A,B). During the ITT, only prebiotic significantly reduced blood glucose (*p* = 0.046; Figure 3A), especially at 90 and 120 min following insulin load (*p* < 0.02) indicating greater insulin sensitivity. During the OGTT, the interaction between RebA and prebiotic influenced blood glucose concentrations at 30 min and 90 min post-glucose load (Figure 3B), with STV higher than all other groups at 30 min and CTR higher than all other groups at 90 min. Although the glucose area under the curve (AUC) was not significantly different between groups, there was a trend towards increased AUC in STV rats compared to CTR rats (*p* = 0.069; Figure 3C).

### 3.4. Gut Permeability Improved by Prebiotic Consumption

DX-4000 FITC concentration was independently influenced by prebiotic (*p* = 0.044; Figure 3D). Lower systemic FITC concentration suggests improved gut barrier integrity with prebiotic.

### 3.5. RebA Consumption Alters Gene Expression in the Mesolimbic Reward System

To investigate the impact of RebA on appetitive behavior, we examined parameters associated with the mesolimbic reward circuit and found that RebA reduced tyrosine hydroxylase (TH) (*p* = 0.044; Figure 3E) and dopamine transporter (DAT) (*p* = 0.044; Figure 3F) mRNA levels in the nucleus accumbens and prebiotic consumption increased TH levels in the ventral tegmental area (*p* = 0.036; Figure 3G).

### 3.6. Gut Microbiota Is Altered by RebA Consumption and Prebiotic Intake

**Figure 4:**
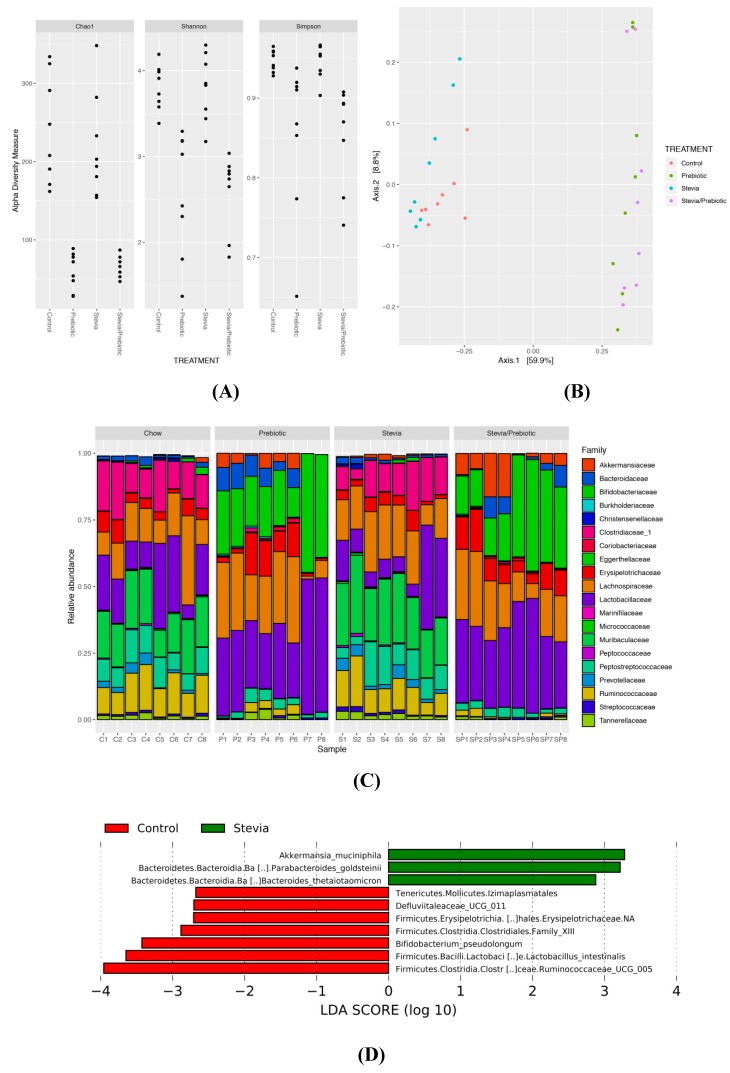
Alpha and beta-diversity and the relative abundance of the most discriminant bacterial groups according to LEfSe in male rats consuming RebA, prebiotics, both or neither for 9 weeks. (**A**) Alpha-diversity measures; (**B**) beta-diversity measures; (**C**) bar plot of microbiota abundance at the family level; (**D**) linear discriminant analysis (LEfSe) describing the greatest differences between stevia (RebA) and control microbial communities; *n* = 8/group.

Prebiotic consumption reduced alpha-diversity (within-sample diversity) (*p* < 0.0005; Figure 4A) and significantly altered beta-diversity (between-sample diversity) (*p* = 0.001; Figure 4B). There was no effect of RebA on alpha- and beta-diversity and no significant interaction between RebA and prebiotic. It is likely that the reduced alpha-diversity in prebiotic samples is a result of the community comprising large abundances of *Bifidobacterium* and *Lactobacillus* (Figure 4C). The relative abundance of *Bifidobacteriaceae* was significantly reduced in STV compared to other groups and was likely driven by the significant increase in species *Bifidobacterium pseudolongum* with prebiotics (Figure S1). Rats consuming prebiotics had significantly greater abundance of these taxa compared to nonprebiotic consumers. Clostridiales family XIII and *Ruminococcaceae UCG 005* showed lower abundance in STV rats compared to CTR and were nearly absent in prebiotic consuming animals. STV animals had increased relative abundance of *Akkermansia muciniphila* and *Akkermansiaceae*, which was further increased in prebiotic rats and showed the greatest abundance in animals consuming STV + PRE. Additionally, STV animals had a greater abundance of *Bacteroides goldsteinii* and *Bacteroides thetaiotaomicron* compared to CTR (Figure 4D).

### 3.7. Prebiotic Reduced Cecal Short Chain Fatty Acid Concentration and RebA Increased Cecal Acetate and Valerate Concentration

**Figure 5:**
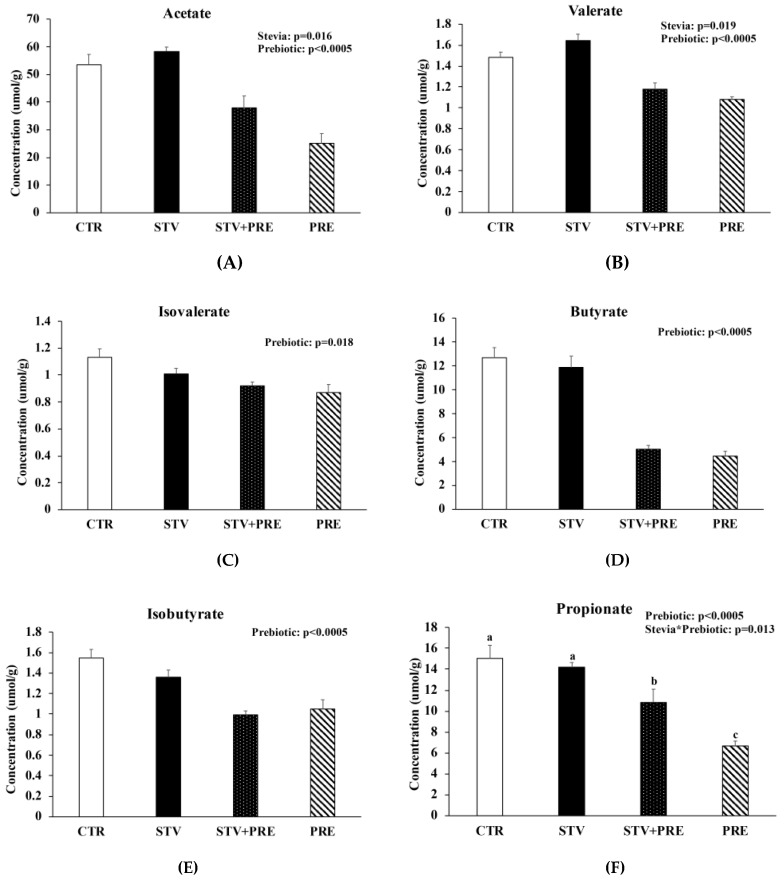
Prebiotics reduced cecal short chain fatty acid concentration and RebA consumption increased acetate and propionate cecal short chain fatty acid concentration. (**A**) Acetate; (**B**) valerate; (**C**) isovalerate; (**D**) butyrate; (**E**) isobutyrate; (**F**) propionate. Values are mean ± SEM, *n* = 8/group. Labelled means without a common superscript letter (a, b, c) differ, *p* < 0.05. CTR, control; STV, RebA; STV+PRE, RebA and prebiotic; PRE, prebiotic.

RebA consumption increased cecal acetate (*p* = 0.016) and valerate (*p* = 0.019) concentration (Figure 5A,B) and prebiotic reduced acetate, valerate, isovalerate, butyrate, and isobutyrate (*p* < 0.02) (Figure 5A–E). Propionate was significantly reduced in STV + PRE and further reduced in PRE animals compared to STV and CTR (*p* < 0.05) (Figure 5F). Acetate showed a positive correlation with fat mass (r_s_ = 0.352; *p* < 0.05) and total weight (r_s_ = 0.466; *p* = 0.01). Similarly, valerate showed a positive correlation with fat mass (r_s_ = 0.456; *p* < 0.011) and total weight (r_s_ = 0.551; *p* = 0.002).

## 4. Discussion

The current study found that young male rats consuming a low dose of Reb A for 9-weeks had altered gut microbiota composition and reduced expression of genes in the nucleus accumbens, a region that plays a key role in food seeking [29]. While prebiotic consumption increased cecal *Akkermansia muciniphila* relative abundance, RebA plus prebiotic consumption increased their relative abundance even further. RebA consumption significantly increased SCFAs acetate and valerate. RebA-associated reductions in the expression of genes in the nucleus accumbens tended to increase when consumed alongside a prebiotic. Additionally, RebA interacted with prebiotics to reduce the markedly increased cecal weight (a marker of increased fermentation) seen with prebiotics alone.

Rats consuming chronic low-dose RebA had an upward glucose AUC trend (*p* = 0.069) during the oral glucose tolerance test and did not show improved insulin sensitivity, as might have been suggested from previous work with higher doses of RebA. Earlier evidence suggested that RebA may reduce hyperglycemia by enhancing insulin secretion and sensitivity [30,31]. Jeppesen et al. (2000) observed insulinotropic effects of steviol and stevioside in perfused islet cells at glucose concentrations between 8.3 mmol/L–16.7 mmol/L [32]. A later study found an acute stevia load (0.2 mg/kg) administered alongside glucose during an intravenous glucose tolerance test increased insulin levels and reduced glucagon levels in a diabetic rat model; changes were not observed in lean rats [33].

Stevia has also been recognized for its potential role in the prevention of obesity by reducing caloric intake. However, one study that found stevia significantly reduced food intake and body weight in female adult rats following 12 weeks of daily consumption, administered stevia doses nearly 100-fold greater than the recommended acceptable daily intake (ADI) [14,34]. Additionally, stevia was administered through drinking water, and authors did not report fluid intake, which may be important to determine whether aversion to the intense sweet taste from a high dose, represented by a decrease in fluid intake, may play a role in reducing food intake [34].

Clinical research by Anton et al. (2010) revealed that lean individuals and those with obesity who consumed a preload containing stevia (290 kcal) 20 min prior to lunch and dinner meals did not compensate by eating more food at meals compared to participants who consumed a preload of sucrose (493 kcal) [30]. Postprandial blood glucose concentrations were significantly lower in subjects consuming a preload of stevia following ingestion of preload compared to subjects who received sucrose or aspartame as a preload meal. Consuming a stevia preload also significantly reduced postprandial plasma insulin levels compared to aspartame and sucrose. Gregersen and colleagues found that fasted diabetic patients who consumed one-gram of stevia in tablet form prior to a test meal had significantly greater glucose tolerance and improved postprandial insulin response and reduced glucagon concentration compared to the placebo group [10]. In many studies, stevia was administered as a single bolus in doses that met and/or exceed the ADI, whereas in the current study, low doses over long-term (9 weeks) were provided and may be one reason we did not observe a similar phenomenon of reduced energy intake or glucose response.

Our results suggest that prebiotic oligofructose-enriched inulin has a more prominent effect on body composition and glucose metabolism than RebA, even when consumed together. Prebiotic fiber has been found to reduce body weight and food intake, liver weight, and plasma glucose concentrations and increase cecum weight in rats [35,36], which aligns with our findings. Further, prebiotic fiber intake has demonstrated a protective role on body weight and glucose metabolism in adverse conditions, like diet-induced obesity and non-alcoholic fatty liver disease [37]. Similarly, prebiotic consumption alongside RebA tended to rescue alterations in gut microbiota composition and mesolimbic reward genes observed from RebA consumption alone.

One mechanism by which prebiotic fiber may exert its protective effects is through modulation of the gut microbiota and subsequent production of short chain fatty acids following fermentation. Prebiotic’s bifidogenic properties and capacity to selectively stimulate growth and proliferation of “health promoting” gut microbes, like *Bifidobacterium*, is well characterized [38]. Prebiotic oligofructose has been shown to improve intestinal permeability, indicated by reduced serum DX-4000-FITC concentration, and may be the result of increased plasma GLP-2, shown to have an intestinotrophic effect [39]. Although we did not examine serum hormones, prebiotics consumed alone and alongside RebA improved gut permeability evident by reduced serum DX-4000-FITC concentration. RebA did not impact intestinal permeability despite its effect on gut microbiota composition.

The mesolimbic reward system plays an important role in food seeking and consists of dopamine neurons originating in the VTA that project to the forebrain structures, including the nucleus accumbens [40]. TH is an enzyme that catalyzes the rate-limiting step of dopamine synthesis and shows decreased expression in leptin-deficient (*ob/ob*) mice in the NAc and VTA alongside reduced evoked dopamine release in the NAc [41]. Alternatively, lower dopamine signaling in obesity leads to decreased physical activity, which may contribute to poorer health outcomes in obesity [42]. Rats consuming RebA had lower TH and dopamine transporter (DAT) mRNA levels in the NAc compared to CTR animals. However, we did not observe any differences in food intake or body composition between these two groups. DAT is responsible for the reuptake of extracellular dopamine into presynaptic neurons [43], and reduced dopamine due to decreased DAT expression uptake has been observed in rats exposed to high fat/sugar diet [44,45]. Additionally, Narayanaswami and colleagues found that obesity-prone rats had decreased DAT expression compared to obesity-resistant animals following exposure to a high-fat diet [46]. Although we did not observe any differences in food and fluid intake between treatment groups, it would be important in future to expose stevia-fed rats to a palatable, high-fat diet to examine if they have a greater propensity to increase food intake, develop obesity, and decrease physical activity as a result of altered TH and DAT gene expression in the nucleus accumbens.

In the current study, we found RebA altered certain microbial taxa compared to the control group, but prebiotics seemed to have a greater impact on gut microbiota composition, even when consumed alongside RebA. RebA consumption reduced members of *Bifidobacteriaceae*, a widely-recognized and well-established “health-promoting” microbiota known for its role in short-chain fatty acid production [47], greater presence in breast-fed infants [48], protection from childhood obesity [49], and in treatment of various diseases through supplementation [50]. However, not all microbiota changes resulting from RebA consumption were negative. We observed that RebA alone increased abundance of *Bacteroides thetaiotaomicron*, which has been shown to induce intestinal angiogenesis through stimulation of Paneth cells, in effect increasing the absorptive capacity of the intestine [51]. Furthermore, RebA consumption reduced the relative abundance of *Lactobacillus intestinalis* to levels similar to prebiotic animals, which was significantly lower than CTR group, despite some *Lactobacillus* strains being found to negatively correlate with change in body weight and fat mass in previous research [52]. However, prebiotic consuming rats had a significant increase in *Lactobacillus* and *Lactobacillaceae*, suggesting that prebiotic only reduced this one particular lactobacilli species. Prebiotic and RebA consumption significantly increased the relative abundance of *Akkermansia muciniphila* compared to all other groups. *A. muciniphila* is a mucin-degrading bacterium and has a lower abundance in obese and diabetic mice [53]. Everard and colleagues found that prebiotic supplementation increased *Akkermansia muciniphila* relative abundance, and consuming *A. muciniphila* as a probiotic improved the aberrant phenotype and metabolic disturbances observed in obese mice [53]. Moreover, *A. muciniphila* treatment was shown to increase lipid oxidation markers and reduced markers of lipogenesis, suggesting a role in fat storage. Therefore, an increased abundance of *A. muciniphila* observed in rats consuming prebiotics and RebA simultaneously may account for the greater reduction in fat mass and improved gut permeability observed in these animals.

RebA consumption increased cecal acetate and valerate SCFA concentration. Acetate is the SCFA produced in the highest molar ratio in the gut and has been implicated in cholesterol synthesis because of its conversion to acetyl coenzyme A by acetyl-CoA synthetase 2 [54,55]; however, this is still controversial [56]. Acetate may also induce lipogenesis in adipose tissue by acting as a ligand for G-protein coupled receptor 43 (GPCR43) and inhibiting lipolysis [57], potentially contributing to an adverse phenotype. Valerate is a branched chain fatty acid and found to be elevated in feces in individuals with obesity [58,59]. Interestingly, we observed a strong positive correlation between cecal acetate and valerate with fat mass and total body weight, of which RebA had greater, but nonsignificant, values. Contrary to what has been shown in other studies [60,61,62], cecal SCFA concentrations were reduced in prebiotic-consuming animals despite increases in *Bifidobacterium* taxa and greater cecal weight. However, one study demonstrated that prebiotic supplementation (fructo-oligosaccharide; FOS) with a nonpurified diet (chow) or semipurified diet (AIN-93) affected cecal SCFA concentrations differently as a result of alternative dietary ingredients in each formula [63]. Authors noted that nonpurified chow contained more nondigestible ingredients, including corn and wheat bran, whereas the semipurified diet contained refined and digestible ingredients, such as casein and corn oil, and may account for the reduced SCFA levels produced when FOS was added to the semipurified versus nonpurified diet [63]. Therefore, our use of a prebiotic-enriched semipurified diet may have led to lower cecal SCFA concentration than normally expected. However, other studies have found that supplementing a purified diet with inulin can increase cecal SCFAs [64]. In future, examining serum SCFA concentrations may provide greater insight into its peripheral actions, and additional research studying this phenomenon is warranted.

Short chain fatty acids are produced from fermentation of carbohydrates and nondigestible foodstuff (i.e., inulin, oligofructose) and are absorbed by colonocytes. Studies have found a positive association between fecal SCFA and central adiposity, body mass index, and serum lipids [65,66], and we observed reduced SCFA concentration in prebiotic animals that had improved body composition. Thus, differences in cecal short chain fatty acid may contribute to altered phenotypes observed between treatment groups.

To our knowledge, this is the first study examining the impact of long-term low-dose stevia consumption beginning in early life in rats and therefore provides important information regarding chronic consumption of this sweetener on glucocentric parameters, body composition, gut microbiota, and the potential rescuing of adverse effects by co-consuming a prebiotic. The strength of the current paper is that RebA doses administered to rats fall within the current adequate daily intake value and may better reflect physiological changes that occur from chronic consumption. Additionally, considering that RebA was administered to rats immediately after weaning, we were able to capture the impact of sweetener exposure that commences in early life on glucocentric parameters, given that there are a growing number of children consuming low-calorie sweeteners. One limitation of the current study is that hormone response during the oral glucose tolerance test was not explored, and therefore, measurements of insulin sensitivity, like the homeostatic model of assessment of insulin resistance (HOMA-IR) could not be quantified. Additionally, incretin hormone response, like GLP-1, could provide valuable information about metabolic responses to glucose and potential mechanism of reduced food intake observed in prebiotic consuming rats. Research examining the long-term impact of consuming stevia is limited, and given its growing popularity in the marketplace, further preclinical and clinical research studying metabolic consequences of chronic stevia intake in various age groups is warranted. Future preclinical research should also provide a metabolic challenge to animals consuming stevia to determine if stevia intake can increase an individual’s predisposition to obesity by increasing intake of palatable foods.

## 5. Conclusions

In conclusion, we found that RebA consumption impacted gene expression in the mesolimbic reward center and certain cecal microbial taxa while prebiotic consumption altered body composition, food intake, glucose tolerance, and cecal microbiota community structure. Additionally, RebA increased SCFAs acetate and valerate, which were positively correlated with fat mass and total weight. Consuming prebiotic alongside RebA tended to mitigate stevia-driven alterations in the mesolimbic reward circuitry and microbiota but attenuated the increase in cecum weight associated with prebiotic intake. Since we observed that RebA reduced the relative abundance of certain “health-promoting” gut microbiota, future studies should examine weight and metabolic outcomes following RebA consumption in populations that exhibit dysbiotic gut microbial compositions (i.e., obese, diabetic). The growing popularity and demand for ‘natural’ low-calorie sweeteners requires greater research examining long-term and low-dose consumption on metabolic and physiological impacts.

## Supplementary Materials

The following are available online at https://www.mdpi.com/2072-6643/11/6/1248/s1, Figure S1: Relative abundance of (A) Bifidobacterium pseudolongum and (B) Akkermansia muciniphila in control, prebiotic, stevia, and stevia+prebiotic consuming rats, Table S1: Composition of experimental diets from age 3- to 8-weeks, Table S2: Composition of experimental diets from age 8- to 12-weeks^1^.
