# Supplementary material for: Low-Dose Stevia (Rebaudioside A) Consumption Perturbs Gut Microbiota and the Mesolimbic Dopamine Reward System
Source: Nutrients. 2019 May 31;11(6):1248. doi: 10.3390/nu11061248 (PMC6627124; doi:10.3390/nu11061248)
Supplement: Supplementary file 1 [file nutrients-11-01248-s001.pdf]

Supplementary Material

Table S1: Composition of experimental diets from age 3- to 8-weeks

(g/kg)	**CTR[1]**	**STV[1]**	**PRE[1]**	**STV+PRE[1]**
Casein	200	200	180	180
Dyetrose	132	132	118.8	118.8
Cornstarch	397.486	397.486	357.737	357.737
Sucrose	100	100	90	90
Cellulose	50	50	45	45
Soybean Oil	70	70	63	63
t-Butylhydroquinone	0.014	0.014	0.0126	0.0126
Mineral Mix	35	35	31.5	31.5
Vitamin Mix	10	10	9	9
L-Cystine	3	3	2.7	2.7
Choline Bitartrate	2.5	2.5	2.25	2.25
Prebiotic	0	0	100	100

[1]Based on AIN93-G diet (Dyets, Inc., Bethlehem, PA). Prebiotic = oligofructose-enriched inulin, Synergy1 (Beneo, Mannheim, Germany). CTR, control; STV, RebA; PRE, prebiotic; STV+PRE, RebA + prebiotic.

Table S2: Composition of experimental diets from age 8- to 12-weeks[1]

(g/kg)	**CTR[1]**	**STV[1]**	**PRE[1]**	**STV+PRE[1]**
Casein	140	140	126	126
Dyetrose	155	155	139.5	139.5
Cornstarch	465.692	465.692	419.123	419.123
Sucrose	100	100	90	90
Cellulose	50	50	45	45
Soybean Oil	40	40	36	36
t-Butylhydroquinone	0.008	0.008	0.0072	0.0072
Mineral Mix	35	35	31.5	31.5
Vitamin Mix	10	10	9	9
L-Cystine	1.8	1.8	1.62	1.62
Choline Bitartrate	2.5	2.5	2.25	2.25
Prebiotic	0	0	100	100

[1]Based on AIN93-G diet (Dyets, Inc., Bethlehem, PA). Prebiotic = oligofructose-enriched inulin, Synergy1 (Beneo, Mannheim, Germany).CTR, control; STV, RebA; PRE, prebiotic; STV+PRE, RebA + prebiotic.

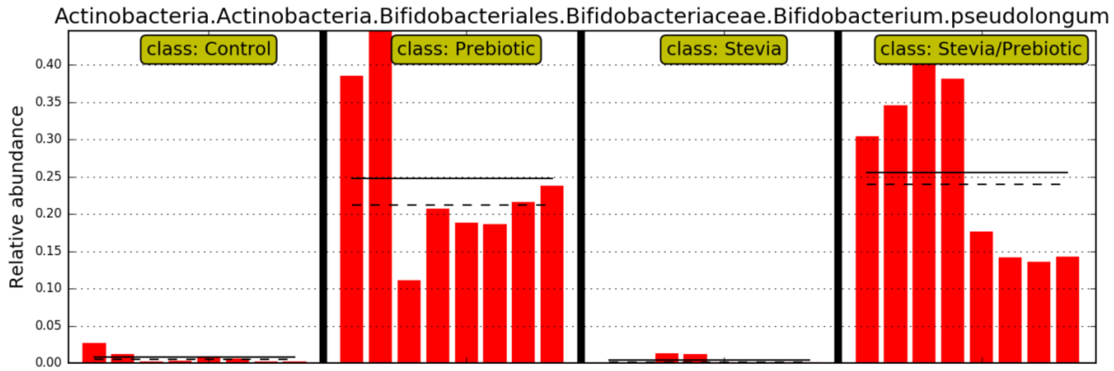

(a)

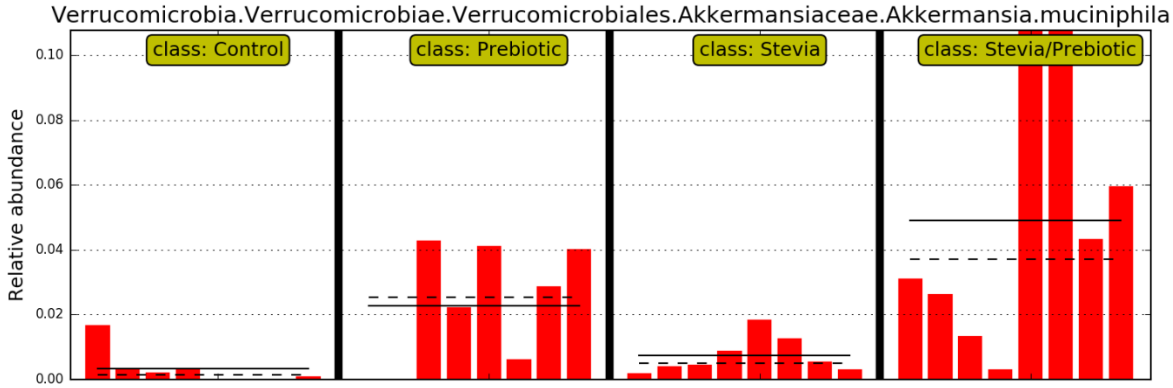

(b)

Figure S1. Relative abundance of (A) *Bifidobacterium pseudolongum* and (B) *Akkermansia muciniphila* in control, prebiotic, stevia, and stevia+prebiotic consuming rats.
